# Animal Chemistry with Reference to the Physiology and Pathology of Man

**Published:** 1845-12

**Authors:** 


Art. IV.—*Animal Chemistry with reference to the Physiology and Pathology of Man.* By Dr. J. Franz Simon, Fellow of the Society for the advancement of Physiological Chemistry at Berlin, etc., etc. Translated and edited by Geo. E. Day, M.A. & L.M., Cantab., Licentiate of the Royal College of Physicians. Philadelphia: Lea & Blanchard. 1845. 8vo. pp. 292. Part 1.

No treatise on physiological chemistry approaches, in fulness and accuracy of detail, the work which stands at the head of this article. It is the production of a man of true

German assiduity, who has added to his own researches the results of the labors of nearly every other inquirer in this interesting branch of science. The death of such a laborer, which is mentioned in the preface to the work as having occurred prematurely in 1843, is indeed a calamity to science. He had hardly reached the middle term of life, and yet had made himself known all over Europe, and in our country, where his name has been familiar for several years as among the most successful of the cultivators of the chemistry of man. We propose to present, as far as our limits will allow, an analytical view of the most striking of the results at which the author arrives in this treatise. We shall look in vain in it for those broad philosophical views with which Liebig captivates his readers; we find in it little of the boldness or originality of the great chemist of Giessen; but, though it be not a system, it is a vast repository of facts, to which the teacher and student may refer with equal satisfaction.

We are not prepared to subscribe to all the author's physiological conclusions. Thus he says in his preface—

"The urea excreted may be regarded as a measure or equivalent of the animal heat developed."

And again:

"Moreover, the amount of carbonic acid and the formation of urea are lessened by a torpid, and increased by an excited circulation; and in proportion to the amount of corpuscles and to the rapidity of the circulation, so much the higher is the animal temperature. Thus in birds we observe a high temperature, and the reverse in the amphibia. In chlorotic, and also in very aged persons, we find a low temperature, and a diminished excretion of urea, while in inflammatory diseases, and after prolonged corporeal exertion the temperature rises, and there is either a relative or an absolute increase of urea; in the former case, even in the absence of all nitrogenous food. The capillary and cutaneous systems tend to regulate an excessive rapidity of the circulation, and to prevent the animal heat from exceeding a certain limit."

Now, while most of what is here said is unquestionably true, and while there does appear to be, in most cases, a certain ratio between the urea and the animal heat, unfortunately for the proposition it is established, that children, in whom the temperature is high, excrete as little urea as men advanced in age whose temperature is correctly stated to be lower than in the adult and middle aged. Lecanu, as the result of 120 analyses of urine, found that the quantity of urea passed in twenty-four hours is in grammes (a gramme is 15.-44 grains troy):

| | Minimum. | Mean. | Maximum. |
|---|---|---|---|
| "By men, - - - - | 23.155 | 28.0525 | 33.055. |
| By women, - - - - | 9.926 | 19.1162 | 28,309. |
| By old men (80 to 86 years,) | 3.956 | 8.1105 | 19.116. |
| By children of 8 years, - | 10.478 | 13.4710 | 16.464. |
| By children of 5 years, - | 3.710 | 4.5050 | 5.300." |

The temperature of women is not lower than that of the rougher sex, and still it seems that the secretion of urea is about fifty per cent. less in them than in men. The declension of this product of secretory action in old age, and its small amount in infancy, are facts of great interest to the physiologist; but the generalization of our author on the subject is not sanctioned by the facts in the case. But it is rather in the multitude of his analysis than in the plausibility of his theories, that his work interests us, and to these we will now ask the attention of the reader.

The translator, Dr. Day, prefixes an account of the mineral and organic constituents of animal bodies, which takes up eighty pages of the volume. We shall not attempt even an enumeration of the various topics embraced in this introduction, but content ourselves with saying, that all the facts which one might wish to find precisely stated may be gathered from it.

The author in the following passage states some of the difficulties of his subject, and the principle that guides the chemist in the analysis of organic matter. Thus—

"The principle upon which these investigations are conducted is dependent on certain questions, which are to be answered by the analysis. Thus in the analysis of the blood, the principal component parts, the water, albumen hæmatin, globulin and fibrin, are usually determined ; but if it be requisite that the analysis should be more fully carried out, we must separate the hæmatin from the globulin, isolate the fats, extractive matters, and salts, and determine their individual proportions. This is the plan that I have usually adopted, and in some cases I have added the determination of sugar, urea, and hæmaphæin. The execution of such a comparatively simple scheme as this is a matter requiring considerable time and labor; and if it were required that we should carry out the analysis still further, and separate the various fats, the different combinations of fatty acids, the varieties of extractive matter, and finally the different salts, our task, in the present state of our knowledge, would be one of great difficulty; and in consequence of the minute proportions in which some of these substances exist in the blood, it would be necessary for us to operate upon a much larger quantity of the fluid than we are usually able to obtain. This method of investigation will probably in a short time be deemed insufficient, for as soon as we have an accurate knowledge of the mode of formation of the extractive matter, its separation and determination will be of the highest importance in explaining many of the phenomena of the metamorphoses of the blood.

"The same is the case with respect to the urine. The formation of a perfect quantitative analysis of this complicated fluid is an extremely difficult (if not an impossible) task, in consequence of the facility with which new products are developed during the progress of the investigation. The course usually pursued has been, therefore, the separation of those constituents which are apparently the most important, the urea, uric acid, salts, and extractive matter; in some cases the estimation of sugar and albumen has been added. The instances in which the separation of the extractive matter into its three principal groups, and the individual analysis of the salts, have been undertaken, are still more rare."

He then refers to the best methods of analysing the several substances, which will be found under the proper heads, but which it will be impossible, if it were desirable, for us to enumerate. The blood is then introduced as the most important of the circulating fluids, and the space devoted to it embraces a mass of facts exceeding in richness anything hitherto presented in our language to the physiologist. First, we have the microscopic analysis of the blood, by which we arrive at the diameter of the blood-corpuscles in man and different animals. The ruminants seem to possess the smallest, in man and monkeys the globules being $\frac{1}{300}$ of a line; in the carnivora they are $\frac{1}{400}$ of a line, while in the ruminantia they are but $\frac{1}{500}$ of a line, equal to 5. The largest blood-globules are in the fishes, among which the torpedo seems to stand at the head of the class. Having given the admeasurement of the globules of the blood, the author next advances to the consideration of the general chemical relations of that fluid. We give only a few examples of change effected in it by chemical reagents. The corpuscles are soluble in caustic ammonia, potash, soda, lime, bile, the acids, alcohol, ether, oil of turpentine, and sulphate of carbon.

"The effects produced by coneia appear, from Hünefield's observations, to be very singular.

"Coneine, either in a state of solution or vapour, reduces the blood to a dirty red greasy mass, which, under the microscope, resembles dark melted wax, and in which no corpuscles can be detected. If diluted blood be treated with a little coneine, it remains fluid, but after a short time, becomes discolored, and throws down a brown sediment. The blood of a rabbit, poisoned with coneine, exhibited no peculiarity."

The formation of the blood is discussed at considerable length, but we pass over the obscure subject to topics promising more satisfactory results. Respiration is one of the functions closely related to the process by which aliment becomes blood. Of the amount of carbonic acid given off by the human subject, of different sexes and ages, we have an estimate in the paragraph annexed:

"It is thus seen that, in general, the amount of carbonic acid expired by both sexes increases with age up to a certain point—the 40–45th year, and then diminishes; that the quantity of carbonic acid expired increases with the development of the muscular system; that women expire less carbonic acid than men; that the formation of carbonic acid attains its maximum at the commencement of menstruation, and then experiences no further increase, except in the pregnant state, until the cessation of menstruation, when an increase again takes place. On an average, an adult male of moderate constitution, exhales from 160 to 170 grains of carbon per hour; an adult female, in the unimpregnated state, from 100 to 110 grains; during pregnancy 125; and after the cessation of the catamenia, from 116 to 130 grains. Dumas also found 154 grains per hour as the average quantity of carbon exhaled by an adult male."

Other circumstances affecting the quantity of carbonic acid expired are the following—

"Hunger and rest diminish, satiety and labor increase it. It is greater during the day than the night, in the proportion of 1·237 to one. If the expired carbonic acid be estimated in relation to the weight of the body, it is found that children give off a proportionally greater amount of this gas than adults. In some forms of disease, the amount of expired carbonic acid falls below the standard; it seems, in a state of health, to vary directly with the activity of the circulation."

The subject of animal heat is closely connected with the circulation of the blood, and the development of carbonic acid. In respect to it the work before us contains its usual rich store of facts, and is sound in its views, the chemists being judges. As to the vital heat of various animals, we are informed, that—

"There is no very great difference between the cetacea and the other mammalia in respect to their temperature. The temperature of the seal and of the Greenland whale has been determined at 104°, and that of the porpoise has been found to vary from 99°·5 to 95°·9. The temperature of the amphibia differs very slightly from the surrounding medium. Czermack found that the temperature of a proteus was 63°·5 when that of the air was 55°·4, was 68°·25 when the temperature of the air was 63°·5, and was 65° in water at 55°; in water of which the temperature was 44°·4, the temperature of a frog was 48°. Dr. Davy found the temperature of a snake 88°·46 in air of 81°·5, and 90° in air of 82°·94; the temperature of testudo midas was 84° while that of the air was 79°·5."

The temperature of fishes, from the experiments of John Hunter and others, is assumed to be 0.7 to 2.7 degrees above that of the surrounding water. These details are followed by the following conclusions:

"It must be regarded as an established fact, that a certain temperature is necessary for the continuance of animal life, and that the source of this temperature must be sought for within the organism, and must be looked upon as a consequence of life itself. The production of heat cannot, however, be so properly ascribed to any of the collective phenomena of life, as to the chemical processes, which are known to develope warmth, and the action of which we see in the metamorphoses; and on the other hand a certain degree of animal heat is indispensably requisite for those chemical processes which are the necessary consequences of the proper organic development of the cells of all tissues, and of their catalytic influence on the nutrient fluid, the plasma of the blood. The animal heat is therefore to be regarded as the product of those vital functions, for the due exercise of which it is essentially requisite. The organism is thus protected against the innumerable disturbing forces under which it would otherwise succumb, in consequence of the varying temperature of the external world. The development of heat, therefore, decreases with the diminution of the vital powers, with the retarded circulation of the blood, with checked nutrition, and with imperfect metamorphosis, while all the phenomena of inanition, perfect destruction of power, and finally an asphyxiated condition, are the consequences.

"As this cellular action, which is collectively exhibited in the metamorphosis of the animal organism, may be regarded as purely chemical, so the heat that is engendered thereby may be considered as a consequence of these chemical processes, and therefore all those functions of the organism which are necessary for the preservation of life, contribute directly or indirectly to the production of animal heat, which must be regarded as developed at every point at which metamorphosis is occurring, and therefore not merely in the lungs but in the whole peripheral system. The absorption of oxygen, and its combination with the carbon of animal matter, not only in the lungs, but in the whole body, must, on that account, be regarded as the principal source of heat. In addition to the oxygen required for the formation of the carbonic acid, a certain amount is absorbed, which probably enters in combination with hydrogen, or with binary or ternary radicals of carbon and hydrogen, of carbon and nitrogen, or of carbon, hydrogen, and nitrogen, and in this manner, doubtless, contributes somewhat to the general production of heat."

The slight independent temperature of the fœtus, and of those young animals that are born in an imperfectly developed condition, the low temperature of persons with morbus cœruleus, and of aged and debilitated persons, the phenomena observed in hybernating animals, all, in the view of this author, support this theory of the source of animal heat. Equally favorable to it he regards the high temperature in inflammatory diseases, when the blood circulates more rapidly than usual, and the metamorphosis is more rapid.

The ordinary constituents of the blood need not be enumerated, but we may mention the substances which are sometimes found, being generally, if not always the result of some morbid action in the system. These are 1. *urea*, which after the extirpation of the kidneys, becomes so abundant that it is easily recognized, as it does also in Bright's disease; 2. *sugar*, a substance perhaps never detected in healthy blood; 3. *bile*, of which the same remark may be made; 4. *fats;* and 5. *extractive matters.*

From the analysis of the author he is led to conclude that

"Arterial blood contains less solid residue generally than venous blood; it contains less fat, less albumen, less hæmatin, less extractive matters and salts than venous blood. The blood-corpuscles of arterial blood contain less coloring matter than those of venous blood."

The blood is affected by constitution; that might be expected. Its constituents must vary with the varying conditions of the system.

"Denis concludes from his analyses that, generally speaking, the stronger the constitution is, the greater will be the amount of solid constituents, and especially of blood-corpuscles. If age is also taken into consideration, my observations confirm those of Denis. At equal ages, the blood in weak constitutions is less abundant in solid constituents and hæmato-globulin than in stronger constitutions."

*Differences dependent on temperament:*

"According to Lecanu, temperament has an influence upon the composition of the blood. He infers from his analyses that the blood of lymphatic persons is poorer in solid constituents, and especially in blood corpuscles, than that of persons of sanguineous temperament, while the quantity of albumen is much the same in both."

*Differences dependent on age:*

"The mass of the blood in the fœtus increases in a very rapid ratio with the development. The proportion of corpuscles is more augmented, and the quantity of water is less than occurs at any subsequent period of life. Even for some time after birth the mass of the blood is relatively large, and the proportion of blood-corpuscles and of iron contained in them is considerably above the ordinary standard.

"Denis has made some experiments on the difference between the blood of very young animals and those of mature age, which confirm the observations already made. His experiments were instituted on dogs.

| | Blood of a dog, 3 months old. | Blood of a puppy, 1 day old. |
|---|---|---|
| Water, - - - - - - | 830.0 | 780.0 |
| Solid residue, - - - - | 170.0 | 220.0 |
| Fibrin, - - - - - - | 2.4 | 2.0 |
| Albumen, - - - - - - | 58.6 | 46.0 |
| Blood-Corpuscles, - - - | 97.0 | 165.0 |
| Extractive matter and salts, - | 12.0 | 7.0 |

"When the skin of the new-born animal loses its red tint, the blood becomes more watery, the blood-corpuscles and the quantity of iron are diminished, and it becomes relatively, but not absolutely, poorer, for its quantity at the same time increases. Subsequently, however, when the generative powers begin to be developed, the corpuscles and the iron increase, and the relative proportion of water diminishes. At the period of full development the excess of corpuscles and iron serves in maintaining the necessary energy of that part of the system, and till the generative powers begin to flag the blood remains abundant in solid constituents, and more especially in corpuscles."

The most important investigation, perhaps, is that which remains to be noticed, and relates to the blood as modified by disease. The author remarks—

"The question whether there exists such a thing as *diseased blood*, is easily answered. The material deviations from its normal condition exhibited by the blood in its physico-chemical relations, in certain morbid conditions of the system, have long been recognised by pathologists."

In a former number we devoted a good deal of space to this subject, as it was brought before the public in the excellent treatise of Andral on hæmatology, and shall attempt to avoid repeating anything given in that article. The composition of healthy blood, as deduced from the mean of our author's analyses, is as follows:

| | |
|---|---|
| "Water - - - - - - - | 795.278 |
| Solid residue - - - - - | 204.022 |
| Fibrin - - - - - - - | 2.104 |
| Fat - - - - - - - | 2.346 |
| Albumen - - - - - - - | 76.600 |
| Globulin - - - - - - - | 103.022 |
| Hæmatin - - - - - - - | 6.209 |
| Extractive matters and salts - - | 12.012" |

And he has found among the specimens of diseased blood, analysed by him, the following variations:

| | | | |
|---|---|---|---|
| "Water - - | may vary from | 888.0 | to 750.0 |
| Solid residue - - | " | 250.0 | 112.0 |
| Fibrin - - - | " | 9.1 | a trace |
| Fat - - - - - | " | 4.3 | 0.7 |
| Albumen - - - | " | 131.0 | 55.1 |
| Globulin - - - | " | 106.6 | 30.8 |
| Hæmatin - - - | " | 8.7 | 1.4 |
| Hæmato-globulin - | " | 115.4 | 31.2 |
| Extractive matters and salts | | 16.5 | 7.6" |

The deviations found by Andral and Gavarret may be given for comparison; they were as follows:

| | | | |
|---|---|---|---|
| "Water - - - | from | 915.0 | to 725.0 |
| Solid residue - - | " | 275.0 | 85.0 |
| Fibrin - - - | " | 10.5 | 0.9 |
| Solid residue of serum | " | 114.0 | 57.0 |
| Blood-corpuscles - | " | 185.0 | 21.0" |

In diseases of an inflammatoıy character this writer found, in common with other observers, that the quantity of fibrin was increased; he found also that the blood had always an alkaline reaction, and states that, according to some experimenters its temperature sometimes attains to 112°.

Proceeding to individual diseases he says:

"The blood usually exhibits the characters of hyperinosis, more decidedly in pneumonia than in most other inflammatory diseases, it also retains its heat for a longer period. The clot is rather below the ordinary size, very consistent, and does not break down for a considerable time. It admits of being sliced, and the sections retain their consistency for some time. Its surface is covered with the buffy coat, and is more or less cupped. The serum is of a pure yellow color. The quantity of solid constituents is usually less than in healthy blood.

"The maximum of fibrin in my analyses was 9.15, which is the largest quantity that I have ever discovered in inflamed blood. The minimum was 3.4, and the mean of four analyses was 6.0. Andral and Gavarret found the maximum of fibrin to be 10.5; the minimum 4; and the mean to fluctuate between 7 and 8. They never met with more than 10.5 of fibrin in the whole course of their analyses."

The following table he copies from Andral and Gavarret, as showing the effect of repeated bleedings upon the constitution of the blood in acute rheumatism:

| Bleeding. | Day of Disease. | Water. | Solid residue. | Fibrin. | Blood-corpuscles. | Solid residue of serum. |
|---|---|---|---|---|---|---|
| 1 | 8 | 778.8 | 221.2 | 6.1 | 123.1 | 92.0 |
| 2 | 9 | 780.9 | 219.1 | 7.2 | 120.7 | 91.2 |
| 3 | 10 | 788.0 | 212.0 | 7.8 | 112.8 | 91.4 |
| 4 | 13 | 799.0 | 201.0 | 10.2 | 101.0 | 89.8 |
| 5 | 17 | 813.9 | 186.1 | 9.0 | 89.2 | 87.9 |
| 6 | 28 | 826.2 | 173.8 | 7.0 | 83.3 | 83.0 |

Dr. Day quotes a case, published by Rindskoff, in which the fibrin mounted up to 12.726. The subject labored under broncho-pneumonia, and was 60 years of age; the blood was taken shortly before his death. In a case of pneumonia biliosa the blood analysed by Scherer gave:

| | |
|---|---|
| "Water - - - - - - | 779.00 |
| Solid constituents - - - - - | 221.00 |
| Fibrin - - - - - - | 9.70 |
| Blood-corpuscles - - - - | 124.60 |
| Albumen - - - - - - | 72.26 |
| Salts - - - - - - - | 9.57 |
| Extractive matters - - - - - | 4.83" |

At a second bleeding it exhibited these proportions:

| | |
|---|---|
| "Water - - - - - - | 785.00 |
| Solid constituents - - - - - | 215.00 |
| Fibrin - - - - - - - | 9.40 |
| Blood-corpuscles - - - - | 122.26 |
| Albumen - - - - - - - | 95.36 |
| Salts - - - - - - - | 8.31 |
| Extractive matters - - - - | 9.67" |

Three days later the patient was again bled, and the blood was found to contain:

| | |
|---|---|
| "Water - - - - - - | 780.00 |
| Solid constituents - - - - - | 220.00 |
| Fibrin - - - - - - - | 12.72 |
| Blood-corpuscles - - - - | 118.47 |
| Albumen - - - - - - - | 69.83 |
| Salts - - - - - - - - | 7.63 |
| Extractive matters - - - - - | 11.35" |

On a fourth venesection it contained:

| | |
|---|---|
| "Water - - - - - - - | 796.00 |
| Solid constituents - - - - - | 204.00 |
| Fibrin - - - - - - - | 8.87 |
| Blood-corpuscles - - - - - - | 106.26" |

Here we must stop, for the present, but we shall resume the subject again. Y.

